# Next Gen Pop Gen: implementing a high-throughput approach to population genetics in boarfish (*Capros aper*)

**DOI:** 10.1098/rsos.160651

**Published:** 2016-12-14

**Authors:** Edward D. Farrell, Jeanette E. L. Carlsson, Jens Carlsson

**Affiliations:** Area 52 Research Group, School of Biology and Environmental Science/Earth Institute, University College Dublin, Belfield, Dublin 4, Republic of Ireland

**Keywords:** fisheries, genotyping by sequencing, microsatellites, next-generation sequencing, population genetics, stock identification

## Abstract

The recently developed approach for microsatellite genotyping by sequencing (GBS) using individual combinatorial barcoding was further improved and used to assess the genetic population structure of boarfish (*Capros aper*) across the species' range. Microsatellite loci were developed de novo and genotyped by next-generation sequencing. Genetic analyses of the samples indicated that boarfish can be subdivided into at least seven biological units (populations) across the species' range. Furthermore, the recent apparent increase in abundance in the northeast Atlantic is better explained by demographic changes within this area than by influx from southern or insular populations. This study clearly shows that the microsatellite GBS approach is a generic, cost-effective, rapid and powerful method suitable for full-scale population genetic studies—a crucial element for assessment, sustainable management and conservation of valuable biological resources.

## 1. Introduction

Understanding the biology and ecology of species and using appropriate monitoring methods are essential for effective assessment, conservation and management. Employing population genetics and genomics to identify populations, stocks or biologically relevant assessment, conservation or management units and determining the degree of genetic exchange between them is an important component of this concept [1,2]. In addition to assessing baseline genetic population structure, it is increasingly recognized that monitoring temporal changes in population genetic metrics can also yield pertinent information and provides a more sensitive and reliable method of establishing demographic parameters and measuring anthropogenic impacts, than traditional monitoring approaches [3].

Population genetics and genomics rely primarily on two types of genetic markers; microsatellites, also known as simple sequence repeats or short tandem repeats (STRs) and single-nucleotide polymorphisms (SNPs). Selectively neutral microsatellites have been the workhorses of population genetics for the past two decades and have played an important role in identifying population structure in numerous species [4,5]. Though, markers under selection are increasingly being included in population genetic studies due to their sometimes higher power to detect contemporary population structure, including microsatellites [6]. Until recently, microsatellites were costly and time-consuming to develop and most studies relied on small numbers of markers [7]. Additional problems associated with microsatellites include fragment size-homoplasy, poor levels of inter-laboratory calibration with genotype based on fragment size rather than the underlying sequence information and laborious genotyping [8–11]. These microsatellite-related problems have led to the increased focus on SNPs [11]. There also remain unresolved problems with SNPs, such as higher susceptibility to ascertainment bias, transferability among SNP genotyping platforms and the requirement for high-quality template DNA [12–14]. The advantages of microsatellites over SNPs for population analyses are the generally higher allelic richness, higher mutation rate and higher statistical power per locus [11,15,16]. It is often perceived that the advantages of microsatellites over SNPs are outweighed by the problems associated with microsatellites, and there is a general shift in favour of using SNPs as a replacement for microsatellites [14].

Next-generation sequencing (NGS) technologies have fundamentally changed the way genetic sequence data are generated and have fuelled a revolution in biological research [17]. NGS has enabled massive increases in the number of sequences attained per sequencing effort and also facilitated rapid and cost-effective genetic marker discovery, including both microsatellites and SNPs [9,13]. NGS has also led to the development of high-throughput genotyping by sequencing (GBS) that can be used for population genetics studies [13,18]. The principal focus of GBS-based methods are SNP genotyping and restriction-site-associated DNA sequencing (RADSeq) [19,20] that allow for simultaneous marker (SNP) detection and genotyping. RADSeq methods [21] generating massive numbers of SNP genotypes per individual can be used to carry out population genetic studies on species with limited, or no, existing sequence data, and have several advantages over previous methods [20]. However, there remains a high individual sample cost for RADSeq-based methods [21] and conservation and management of natural populations requires accurate and inexpensive genotyping methods [9]. Furthermore, it is important to remember that many questions in molecular ecology can be efficiently addressed with a limited number of highly polymorphic markers, such as microsatellites [5]. Many of the issues associated with microsatellite-based population studies could be mitigated using an NGS-based microsatellite GBS approach leading to faster and cheaper genotyping in large-scale population genetics studies while offering better and more data than conventional methods [22,23].

Vartia *et al*. [22,24] and later Darby *et al*. [23] demonstrated the potential of NGS for microsatellite GBS using de novo and existing capillary/gel electrophoresis multiplex marker panels and validated the GBS approach against microsatellite data generated by traditional capillary electrophoresis. Vartia *et al*. [22] and Darby *et al*. [23] also demonstrated that the GBS approach detected a greater number of unique alleles than capillary electrophoresis and resulted in the ability to resolve greater population genetic structure. In addition, Vartia *et al*. [22] introduced a method of combinatorial barcoding to enable pooling of samples in large-scale population genetics studies without the need for multiple library preparations or multiple sequencing runs. However, neither study applied the method on a large scale nor used the full potential of an NGS run.

The current study aims to use a modified protocol from Carlsson *et al*. [7] for marker discovery and an improved version of the Vartia *et al*. [22] method for GBS to demonstrate a rapid and cost-effective method for conducting a large-scale de novo population genetics study in a non-model species, boarfish (*Capros aper*, L.).

The boarfish is a small marine pelagic shoaling species distributed in shelf waters from Norway to Senegal, including the Mediterranean and oceanic island waters [25]. Boarfish in the northeast Atlantic area are a long-lived species that reach a maximum age of more than 30 years with a length- and age-at-maturity of 9.7 cm total length (TL) and 3.4 years, respectively [26,27]. In the northeast Atlantic, boarfish have historically been considered rare and reported to undergo periodical fluctuations in abundance with recent increases observed in the Bay of Biscay, Galician continental shelf and the Celtic Sea between the 1980s and 2000 [25,28,29]. These increases in abundance have been tentatively attributed to enhanced adult growth and recruitment as a result of climate-related changes in environmental conditions [25,30]. As a result of the apparent increase in abundance a target pelagic trawl fishery developed in shelf waters southwest of Ireland [31].

Analyses of bottom trawl survey data suggested a hiatus in distribution of boarfish between the northern Spanish Shelf and Portuguese waters [31]. More recently, apparent differences in the reproductive characteristics of *C. aper* in the northeast Atlantic region [26] and within Portuguese waters [32] have been reported. For assessment and management purposes, a single northeast Atlantic ‘stock’ was considered to exist north of Portuguese waters (ICES Subareas 6, 7 and 8) and the current management strategy only considers the ‘stock’ in this area. It is not known if this ‘stock’ comprises a single or multiple biological populations and the delineation of the ‘stock’ boundaries remain uncertain. The connectivity among elements of this purported ‘stock’ with boarfish to the south, in the Mediterranean Sea and isolated insular boarfish elements remains unknown. It also unclear if the reported recent increases in abundance of boarfish [25,28,29] are a result of population expansion within the northeast Atlantic or an influx of boarfish from southern or oceanic regions. In order to ensure the accurate assessment and sustainable management of the species these outstanding issues must be resolved.

The current study aims to resolve this by employing NGS to undertake a modified marker discovery protocol from Carlsson *et al*. [7] and an improved version of the Vartia *et al*. [22] microsatellite GBS method to:

(i) develop a de novo suite of informative microsatellite markers for boarfish,
(ii) assess the genetic population structure of boarfish across the species' range, and
(iii) investigate if purported recent increases in abundance in the northeast Atlantic area are the results of an influx from other regions.

## 2. Material and methods

### 2.1. Sampling and DNA isolation

Samples of boarfish were collected from the catches of fisheries surveys and commercial fishing operations from across the species' range. Each fish was measured for TL to the 1.0 mm below and total body weight to the nearest 0.01 g. The body cavity was opened with a semi-circle incision running anteriorly from the cloaca to the pectoral fin to assess sex and maturity. Where it was not possible to determine the sex based on visual inspection the specimen was recorded as unsexed (U). A 1 cm^3^ piece of tissue was excised from either the gills or the caudal peduncle of each specimen and stored at 4°C in absolute ethanol. Total genomic DNA (gDNA) was extracted from 10 mg of tissue from each fish using a chloroform/isoamyl alcohol protocol. Extracted DNA was quantified on a NanoDrop® ND-1000 spectrophotometer (Nano-Drop Technologies, Wilmington, DE, USA), standardized to 40 ng µl^−1^ and laid out on 96-well PCR plates.

### 2.2. Microsatellite discovery and primer/combinatorial barcode design

**Figure 1.**
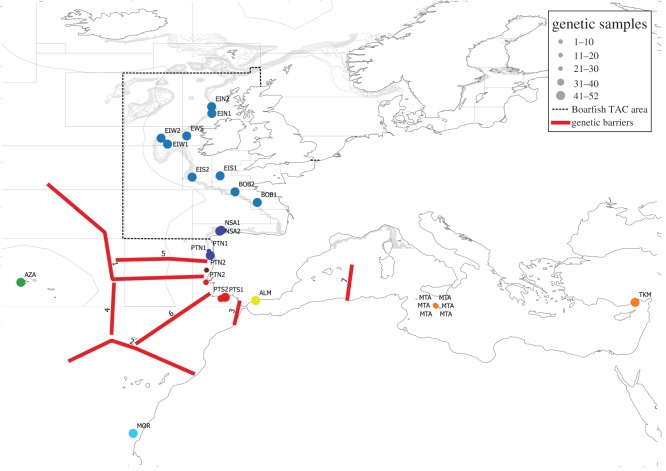
Map of the labelled sampling locations. Locations are colour coded according to clustering analyses. Genetic barriers are illustrated in red and numbered sequentially. The current northeast Atlantic boarfish stock management area and ICES areas are also indicated.

Two specimens, one from the northwest of Ireland (EIN2) sample and one from the Alboran Sea (ALM) sample (figure 1) were selected for microsatellite discovery through shotgun sequencing. Each sample comprised muscle tissue from the caudal peduncle and yielded high-quality gDNA which was considered to be free from parasitic contamination. The samples were also considered likely to be from genetically differentiated populations due to isolation by geographical distance. Isolated gDNA (50 µl at 50 ng µl^−1^) was sent to the Duke Center for Genomic and Computational Biology (GCB, Duke University, USA) for library preparation and shotgun sequencing. Genomic DNA from the two samples was fragmented to 300 bp insert size and two libraries were prepared with the KAPA Hyper Prep Kit (Kapa Biosystems Ltd). The libraries, one per individual, (10 pM with 5% PhiX) were sequenced on an Illumina MiSeq Platform (Illumina Inc.) with a 500-cycle MiSeq Reagent Kit V2 to yield 250 bp paired-end (PE) reads.

Raw FASTQ sequence data were downloaded from Illumina BaseSpace and initial quality control was performed using FastQC [33]. Overlapping R1 and R2 PE reads were assembled and exported in FASTA format with PANDAseq paired-end assembler for illumina sequences [34]. Assembled reads containing microsatellites were identified and extracted with QDD_pipe 1: microsatellite detection in QDD-VM v. 3.2.1 [35,36]. Microsatellite-containing sequences were subsequently assembled with MIRA 4.0 and resulting contigs converted to .ACE file format using MIRA Convert [37]. Assembled reads were imported into Tablet [38] and visually analysed to identify polymorphic microsatellite loci. Assemblies containing polymorphic loci were imported into Geneious® 7.0 [39] and the corresponding consensus sequences generated. Consensus sequences were screened for redundancy, to ensure that the same microsatellite loci were not detected in multiple consensus sequences, by performing a de novo assembly in Geneious® 7.0 using default settings.

Locus-specific forward and reverse primers were designed for putatively polymorphic microsatellite loci with the Primer3 application [40] in Geneious® 7.0 with optimal primer length set at 20 bp, optimal *T*_m_ at 60°C and product size range at 120–180 bp. Primers were designed to bind in conserved flanking regions to minimize the possibility of null alleles. Primers were cross-referenced with the original sequence dataset to identify primers that annealed to multiple regions, which if detected were excluded. The forward and reverse locus-specific primers were adapted, to facilitate combinatorial barcoding of amplicons, by adding either an M13-R (5′-GGAAACAGCTATGACCAT-3) or CAG (5′-CAGTCGGGCGTCATCA-3′) universal tail to the 5′ end as described by Vartia *et al*. [22]. The modified primers were tested for the formation of secondary structures (hairpins, primer dimers and hetero dimers) with the IDT OligoAnalyzer Tool 3.1 (http://eu.idtdna.com/calc/analyzer) and were ordered as 100 µM stock solution (IDT, Leuven, Belgium). Multiplex panels were generated in MultiPLX 2.1 [41] using the low grouping stringency setting and the maximum number of primers per group set at 20. Primers were diluted to 10 µM working solution and combined according to the MultiPLX 2.1 output to form five 0.25 µM multiplexes.

A set of 96, 11 bp combinatorial barcodes suitable for amplicon sequencing on the Illumina MiSeq platform were designed based on the 12-bp Golay-barcodes from Caporaso *et al*. [42], following Vartia *et al*. [22]. The last base of the Caporaso *et al*. [42] barcodes was removed and an M13-R universal tail was added to the 3′ end of 48 of the barcodes and a CAG universal tail to the 3′ end of the remaining 48 barcodes (electronic supplementary material, table S1), yielding 2304 possible combinations. The modified barcodes were tested for the formation of secondary structures (hairpins, primer dimers and hetero dimers) with the IDT OligoAnalyzer Tool 3.1 (http://eu.idtdna.com/calc/analyzer) and ordered as 100 µM stock solution (IDT). Barcodes were diluted to 1 µM working solution and laid out on 96-well PCR plates; M13-R-tailed barcodes were arranged A–H and CAG-tailed barcodes were ordered 1–12.

### 2.3. Amplicon generation, barcoding and pooling

Microsatellite amplification reactions were carried out using a two-step PCR. Step 1 had a total amplification reaction volume of 10 µl and contained 5 µl of 2X Multiplex PCR Master Mix (Qiagen, Hilden, Germany), 2.5 µl of ddH_2_O, 1.5 µl of gDNA (40 ng µl^−1^) and 1 µl of multiplex primers (0.25 µM). Step 1 amplifications were performed in a Biometra TProfessional Thermocycler with lid temperature of 95°C, using a thermal cycling profile of initial heating of 95°C for 15 min, followed by 30 cycles of 94°C for 30 s, 60°C for 1.5 min, and 72°C for 1 min, followed by a final extension step of 60°C for 30 min. Completed reactions were paused at 4°C before initiating Step 2, which involved the incorporation of barcodes into the amplicons. PCR plates were spun briefly and carefully opened. A volume of 1 µl of the relevant M13-R tailed barcode (1 µM) and 1 µl of the relevant CAG-tailed barcode (1 µM) were pipetted into each well. PCR plates were resealed with strip caps before vortexing and centrifuging briefly. A thermal cycling profile of initial heating of 95°C for 15 min, followed by eight cycles of 94°C for 30 s, 53°C for 1.5 min, and 72°C for 1 min, followed by a final extension step of 60°C for 30 min, was then used to anneal barcodes to amplicons. The two individual specimens used for microsatellite discovery in the shotgun sequencing run were included in the amplicon sequencing run as controls to enable analysis of the concordance in genotype calls between the sequencing efforts.

In order to pool each plate of amplicons (i.e. 96 samples amplified at 1 multiplex), 9 µl from each well was pipetted into a new 1.5 ml Eppendorf tube. Pooled amplicons were stored at 4°C until all plates of amplicons were generated. The pooled amplicon samples were visualized on a 2% agarose gel to confirm the presence of the expected fragment lengths and to confirm barcode incorporation into amplicons. Subsequently, 40 µl of each of the pooled amplicon samples was purified with ExoSAP-IT® (Affymetrix UK Ltd, UK) following manufacturer's protocol. The concentration of the purified pooled amplicon samples was measured on a Qubit® Fluorometer (Invitrogen, ThermoFisher Scientific) using the Qubit® dsDNA HS Assay Kit (Invitrogen, ThermoFisher Scientific). Pooled amplicon samples were standardized and 400 ng of each amplicon was added to a new 1.5 ml Eppendorf tube. The concentration of the combined sample was measured on a Qubit® Fluorometer and diluted to 50 ng µl^−1^ for library preparation and amplicon sequencing at Duke Center for Genomic and Computational Biology (GCB, Duke University). A single library was prepared with the KAPA Hyper Prep Kit (Kapa Biosystems Ltd) and 10 pM with 5% PhiX was sequenced on an Illumina MiSeq Platform (Illumina Inc.) with a 600-cycle MiSeq Reagent Kit V3 to yield 300 bp paired-end (PE) reads.

### 2.4. Sequence sorting and genotyping

Raw FASTQ sequence data were downloaded from Illumina BaseSpace and initial quality control was performed using FastQC [33]. Reads were sorted and grouped using a modified python script [22] based on the Levenshtein distance metric, which measures the distance between two sequences of characters. In short, the raw sequence data were processed by identifying sequence reads containing the forward and reverse (combinatorial) barcodes and the locus-specific primers. The python script was set to allow for zero errors in either the combinatorial barcodes or primers. Reads were sorted hierarchically and grouped into five separate FASTA files as reads with: no barcode, one barcode, two barcodes and no primers, two barcodes and two non-matching primers, two barcodes and two matching primers.

Only reads containing two barcodes and two matching primers were included in further analyses. These reads were grouped by locus and individual before removing the barcode from the sequences. Grouped reads were imported into Geneious® 7.0 in FASTA format and organized into folders per locus per individual. Loci were manually genotyped by viewing all of the reads of a particular individual at a specific locus, as a read length histogram and verified by read alignment [22]. Loci that were not polymorphic, had poor amplification success or were confounded by a high level of stutter were not genotyped. Of the successfully amplified loci, only individuals with 10 or more reads and unambiguous genotypes for a given locus were genotyped.

### 2.5. Statistical analyses

Individuals and loci that had less than 75% genotyping success were excluded from the dataset in order not to bias the analysis. The software MICRO-CHECKER 2.2.3 [43] was used, under default settings, to identify possible genotyping errors, including stuttering, large allele drop-outs and null alleles. Deviations from Hardy–Weinberg equilibrium (HWE), linkage disequilibrium (LD) and excess and deficiency of heterozygotes were tested with Genepop 4.2—default settings [44]. Microsatellite Analyzer (MSA) 4.05 [45] was used, under default settings, to assess the number of alleles, allelic richness, allele size ranges, expected and observed heterozygosities, multi-locus global and pairwise *F*_ST_ estimates and Nei's *D*_A_ distance with 1000 bootstrap replications [46]. In all cases with multiple tests, significance levels were adjusted using the sequential Bonferroni technique [47].

LOSITAN was used to detect loci that potentially showed footprints of selection [48]. LOSITAN was run with both the infinite allele (IA) and stepwise mutation model (SMM) with each run comprising 100 000 simulations with both ‘Neutral mean *F*_ST_’ and ‘Force mean *F*_ST_’ selected, a confidence interval of 0.95 and a false discovery rate of 0.1. For the initial LOSITAN run, the data were divided into the original 20 samples. Subsequently pairwise runs were performed using groupings informed by the clustering analyses described below in order to detect on what geographical scale footprints of selection could be operating.

POWSIM v. 4.1 [49] was used to estimate whether the number of loci and their allele frequencies provided sufficient statistical power to detect significant genetic differentiation. Simulations were run using default parameter values for dememorizations (1000), batches (100) and iterations per batch (1000), and a range of different values of *F*_ST_ (0.0005–0.0488) were tested by varying the number of generations of drift (*t*) while keeping the effective population size (*N*_e_) constant at 1000. The statistical power was estimated after 1000 replicates as the proportion of statistically significant tests (*p* < 0.05). The probability of obtaining false positives when the true *F*_ST_ = 0 was also obtained at generation *t* = 0 as a measure of *α* error rate.

ARLEQUIN 3.5.1.3 [50] was used to perform analysis of molecular variance (AMOVA, default settings) in order to partition the total observed variance into between-years variability to estimate temporal stability, and between-locations variability to estimate the spatial structure and compare variability between and among groupings.

STRUCTURE 2.3.4 [51,52] was used to estimate the number of clusters, *K*, present among the samples. The STRUCTURE analysis was conducted on the full 20 sample dataset using the admixture ancestry model, correlated allele frequencies, a burn-in period of 10^5^ iterations followed by 10^6^ MCMC steps and *K* values from 1 to 20 with five replicates of each. Structure runs were combined using Structure Harvester v. 0.6.94 [53] and the most likely *K* was assessed by estimating and plotting ln *P*(*D*) and implementing the delta *K* method if necessary [54]. CLUMPP [55] was used to align cluster assignment across the replicates and plots of the clusters were produced in Microsoft Excel.

In order to identify potential barriers to gene flow among geographical sampling locations Monmonier's maximum-difference algorithm [56], implemented in BARRIER 2.2 [57], was employed to analyse 1000 bootstrapped Nei's *D*_A_ distance matrices from the MSA analysis. The significance of barriers was determined by analysis of the bootstrapped datasets following the method in [57].

The directional relative migration between inferred clusters was estimated using the divMigrate function [58] from the R-package DIVERSITY [59], using Jost's *D* [60], *G*_ST_ [61] and *Nm* [62] as measures of genetic distance. To test whether migration between clusters was asymmetrical (significantly higher in one direction than the other), 95% confidence intervals were calculated from 10 000 bootstrap iterations.

The current study aimed to discover and validate an excess of microsatellite loci in order to ensure that sufficient statistical power was available to detect population structure. In order to test how the number of employed loci affected the accuracy of estimating global multi-locus *F*_ST_, datasets were generated by randomly selecting 5, 10, 15, 20, 25, 30, 32, 35 or 40 loci with each condition (number of loci) replicated 10 times and analysed for global multi-locus *F*_ST_ using GENETIX 4.05 [63]. Average global multi-locus *F*_ST_ and 95% confidence interval of the 10 replicates were calculated and plotted to visualize the variability of average *F*_ST_ estimates as a function of numbers of loci.

Furthermore, the potential for high-grading microsatellite loci, in order to reduce the number of loci required for future monitoring of boarfish population dynamics, was also explored. To facilitate high-grading, the available temporal replicate samples were separated into those collected in 2010/2011 and those collected in 2013/2014 (table 1). The separation of temporal samples will reduce the occurrence of false positives (loci behaving stochastically, indicating high levels of genetic differentiation while not temporally stable—false positives). Pairwise single-locus *F*_ST_ was estimated using groupings informed by the clustering analyses to elucidate if a subset of high-graded loci were temporally stable and able to differentiate the same population structure as the full marker set.

**Table 1.** Boarfish sample collection details.

code	sample label	vessel	date	area	lat	lon	no. (M/F/U)	length M/F/U (mm TL)
ALM	ALM001-ALM050	RV Cornide de saavedra	5 May 2011	Mediterranean - Alboran Sea	36.6500	−4.5000	50 (0/3/47)	n.a./62–69/47–67
AZA	AZA001-AZA050	n.a.	14 Dec 2011	Azores	38.5200	−28.6200	50 (1/3/46)	120/75–105/65–102
BOB1	BOB001-BOB046	RV Thalassa	2 Nov 2010	Bay of Biscay	46.7000	−4.3300	46 (14/17/15)	70–95/70–95/65–95
BOB2	BOB047-BOB100	MFV Vigilant	5 Mar 2013	Bay of Biscay	47.7833	−6.6167	54 (29/24/1)	85–94/83–97/85
EIN1	EIN001-EIN050	RV Celtic Explorer	29 Sep 2011	Malin shelf	55.8200	−8.9900	50 (18/32/0)	112–147/127–166/n.a.
EIN2	EIN051-EIN100	RV Celtic Explorer	1 July 2013	Malin shelf	56.5178	−8.9375	50 (25/25/0)	125–151/130–160/n.a.
EIS1	EIS001-EIS050	MFV Felucca	21 July 2011	Celtic Sea shelf	49.4300	−8.1500	50 (25/25/0)	105–135/105–160/n.a.
EIS2	EIS051-EIS100	MFV Felucca	23 July 2013	Celtic Sea shelf	49.2998	−11.0220	50 (26/24/0)	122–148/92–163/n.a.
EIW1	EIW001-EIW050	MFV Felucca	10 July 2011	Porcupine Bank	52.6700	−13.5500	50 (25/25/0)	124–153/128–170/n.a.
EIW2	EIW051-EIW1000	MFV Felucca	11 July 2013	Porcupine Bank	53.2800	−14.2100	50 (25/25/0)	131–157/137–169/n.a.
EWS	EWS001-EWS050	MFV Felucca	13 July 2013	West of Ireland	53.5200	−11.5800	50 (30/20/0)	105–160/109–165/n.a.
MOR	MOR001-MOR050	RV Dr. Fridtjof Nansen	22 Nov 2011	Western Sahara	23.0300	−17.0800	50 (21/29/0)	86–116/73–136/n.a.
MTA	MTA001	MFV Ignazio Padre	19 Oct 2011	Mediterranean - Malta	36.0693	14.0033	1 (1/0/0)	102/n.a./n.a.
MTA	MTA002	MFV Ignazio Padre	15 Mar 2012	Mediterranean - Malta	36.1539	13.9431	1 (1/0/0)	98/n.a./n.a.
MTA	MTA003-MTA011	MFV Ignazio Padre	29 Mar 2012	Mediterranean - Malta	36.1051	14.0225	9 (1/8/0)	97/89–102/n.a.
MTA	MTA028-MTA033	MFV Ignazio Padre	22 May 2012	Mediterranean - Malta	36.1065	14.0484	6 (0/6/0)	n.a./80–130/n.a.
MTA	MTA012-MTA015	MFV Ignazio Padre	29 May 2012	Mediterranean - Malta	36.0713	14.0019	4 (2/2/0)	87–88/75–95/n.a.
MTA	MTA016-MTA027	MFV Ignazio Padre	29 May 2012	Mediterranean - Malta	36.1658	13.9372	12 (0/12/0)	n.a./82–95/n.a.
MTA	MTA047-MTA050	MFV Ignazio Padre	05 July 2012	Mediterranean - Malta	36.1697	13.9224	4 (1/3/0)	90/82–95/n.a.
MTA	MTA034-MTA046	MFV Ignazio Padre	05 July 2012	Mediterranean - Malta	36.0612	14.0201	13 (2/11/0)	90–98/83–100/n.a.
NSA1	NSA001-NSA050	RV Thalassa	6 Apr 2011	Northern Spanish Shelf	43.8500	−8.0400	50 (30/20/0)	113–155/95–163/n.a.
NSA2	NSA051-NSA100	RV Miguel Oliver	21 Mar 2014	Northern Spanish Shelf	43.7592	−8.1841	50 (23/27/0)	111–157/113–161/n.a.
PTN1	PTN001-PTN006	RV Noruega	1 Oct 2011	Northern Portuguese shelf	41.7167	−9.3017	6 (5/1/0)	107–144/150/n.a.
PTN1	PTN008-PTN050	RV Noruega	2 Oct 2011	Northern Portuguese shelf	41.2650	−9.1633	43 (19/24/0)	112–161/108–165/n.a.
PTN2A	PTN051-PTN065	RV Noruega	6 Oct 2013	Northern Portuguese shelf	39.7700	−9.5400	15 (0/0/15)	n.a./n.a./69–82
PTN2B	PTN066-PTN092	RV Noruega	6 Oct 2013	Portuguese shelf	38.5200	−9.6000	27 (11/10/6)	113–153/115–144/66–111
PTS1	PTS001-PTS050	RV Noruega	21 Oct 2011	Gulf of Cadiz	36.9800	−7.6200	50 (7/19/24)	78–85/62–82/58–85
PTS2	PTS051-PTS090	RV Noruega	17 Oct 2013	Gulf of Cadiz	36.8517	−8.0567	40 (1/1/38)	102/104/59–91
TKM	TKM001-TKM041	RV Lamas	22 Apr 2011	Mediterranean - Turkey	36.4635	34.4662	41 (4/12/25)	60–65/62–79/26–74

## 3. Results

### 3.1. Sampling and DNA isolation

In total, 20 samples comprising 972 boarfish were collected from across the distribution range (figure 1 and table 1) with a ratio of males to females of 1 : 1.2. Temporal sampling (samples from two different years) was achieved in samples collected within the current management area and in Portuguese waters, while only one temporal sample per location was collected from the extremities of the species' range. Total gDNA was successfully extracted from a total of 960 boarfish and laid out on ten 96-well PCR plates following standardization.

### 3.2. Microsatellite discovery and primer/combinatorial barcode design

Shotgun sequencing of two specimens yielded 15 090 710 reads (8 398 530 from EIN2 and 6 692 180 from ALM) with an average Q30 more than 90%. Paired-end assembly of the raw reads in PANDAseq resulted in 5 785 900 paired-end reads. QDD-VM v. 3.2.1 detected 2 207 651 microsatellite-containing sequences in the paired reads. MIRA assembled 450 520 of these reads into 34 666 microsatellite-containing contigs with a length range of 78–3609 bp and comprising between 2 and 897 reads. Two hundred contigs, comprising 10–79 reads, containing putatively polymorphic microsatellite loci, with a read depth per locus ranging from 2 to 40, were selected following visual analysis in Tablet. The initial 200 loci were reduced to 85 microsatellite loci following screening for redundancy in Geneious® and secondary structure testing with the IDT OligoAnalyzer Tool 3.1. The corresponding 85 primer pairs were divided into five multiplex panels, each comprising 17 primer pairs, in MultiPLX 2.1 (electronic supplementary material, table S2).

### 3.3. Amplicon generation, pooling and sequencing

The 10 plates of gDNA, amplified at five multiplexes, resulted in fifty 96-well plates of amplicons for pooling. After pooling, the final sample for sequencing comprised the pooled amplicons of 960 boarfish at 85 microsatellite loci or 81 600 genotypes. Amplicon sequencing of the single library yielded 17 979 084 raw reads with an average Q30 more than 65%, 841 K mm^−2^ cluster density and a GC content of 46%.

### 3.4. Sequence sorting and genotyping

In total, 3 776 251 (21%) reads were identified as having two matching barcodes and two matching primers and were successfully assigned to a specific individual and locus (table 2). The remaining 14 202 833 reads were affected by a number of issues including having only one identifiable barcode (54.1%), no identifiable barcodes (23.6%), two barcodes and two non-matching primers (1.1%) and two barcodes and no identifiable primers (0.3%). Of the 85 putative polymorphic loci analysed, 42 produced scorable amplicons and were successfully genotyped, 13 were not polymorphic, 15 were confounded by a high level of stutter, 14 had poor amplification success and low read depth, and one did not amplify (electronic supplementary material, table S2). The number of reads recovered for the 42 genotyped loci ranged from 19 489 to 86 627 with an average of 52X reads per individual and locus (electronic supplementary material, table S3). The number of individuals with less than 10 reads at a particular locus ranged from 23 to 231 (2–24% of total), and these were not genotyped at that locus (electronic supplementary material, table S3). On average, 91% of individuals amplified at each of the genotyped loci. In total, 121 individuals were genotyped at less than 75% of loci and were not included in further analyses (electronic supplementary material, table S4). Analysis of the two control individuals, which were sequenced in both the shotgun and amplicon sequencing efforts, confirmed that all alleles present in the shotgun sequencing effort were also detected in the amplicon sequencing effort. Additional alleles were detected among the sequenced amplicon's which is probably due to the lower read depth of the shotgun effort.

**Table 2.** Breakdown of recovered reads from amplicon sequencing MiSeq run.

read grouping category	no. reads	% of total
two barcodes and two matching primers	3 776 251	21.0
two barcodes and two non-matching primers	190 521	1.1
two barcodes and no primers	45 165	0.3
one barcode	9 730 410	54.1
no barcode	4 236 737	23.6
total	17 979 084	100

### 3.5. Genetic variability

MICRO-CHECKER analysis indicated that genotyping was not affected by technical artefacts, errors or large allele drop-outs; however, two loci were identified as potentially affected by null alleles: *BOF209* and *BOF401*. Both of these loci also displayed significant deviations from HWE, in 12 and six samples, respectively, and potential LD in one sample (*NSA1*) and as such were removed from further analyses (electronic supplementary material, table S5). Of the remaining 40 loci, 11 had indications of deviations from HWE in less than or equal to 4 out of 20 samples each. However, there was no discernible pattern of deviations among samples (electronic supplementary material, table S5). Therefore, these 40 loci and all 20 samples were retained for further analyses. Potential LD was also indicated in the *BOB2* sample between the loci *BOF417* and *BOF511*. However, this linkage was not indicated in any other samples. It is likely that the loci were affected by gametic phase disequilibrium and not actually physically linked and thus they were retained in the subsequent analyses. The allelic richness (*Rs*) per locus and sample ranged from 1.64 at locus *BOF507* in *MOR* to 13 at locus *BOF304* in *TKM*. Average observed heterozygosity (*H*_O_) ranged from 0.02 at locus *BOF507* in *MOR* to 0.95 at locus *BOF202* in *PTN1*.

The initial LOSITAN run comprising the full 20 sample dataset identified the loci *BOF211*, *BOF214*, *BOF305* and *BOF507* as outliers potentially under directional selection under both the IA and SMM models and loci *BOF404* and *BOF417* as outliers potentially under balancing selection under both models (electronic supplementary material, table S6). The IA model also indicated the loci *BOF116* and *BOF514* as outliers potentially under balancing selection. To test for potential effects of the outlier loci, the initial POWSIM, MSA and STRUCTURE analyses were conducted using (i) all 40 loci and all 20 samples, (ii) using 32 loci (excluding the eight outlier loci) and all 20 samples.

POWSIM results for both the 40 and 32 loci datasets indicated that the probability of detecting population structure was high, and statistically significant at *F*_ST_ ≥ 0.001 for *χ*^2^ test and *F*_ST_ ≥ 0.0025 for Fisher's test (table 3). When *F*_ST_ was set to zero (which simulates no divergence among samples), the proportion of falsely significant values (*α* type I error) was lower than the intended value of 5% for *χ*^2^-test and was 7% for Fisher's test.

**Table 3.** POWSIM results for the 32 and 40 loci datasets.

no. loci	expected *F*_ST_	χ^2^	Fisher's test	*T*
32	0	0.045	0.070	0
32	0.0005	0.511	0.477	1
32	0.001	0.949	0.893	2
32	0.0025	1.000	1.000	5
32	0.005	1.000	1.000	10
32	0.01	1.000	1.000	20
32	0.0488	1.000	1.000	100
40	0	0.034	0.073	0
40	0.0005	0.592	0.508	1
40	0.001	0.986	0.938	2
40	0.0025	1.000	1.000	5
40	0.005	1.000	1.000	10
40	0.01	1.000	1.000	20
40	0.0488	1.000	1.000	100

The global multi-locus *F*_ST_ for the 40 loci was estimated at 0.0245 (0.0159–0.0364 95% CI) and for the 32 loci was 0.0192 (0.0147–0.0239 95% CI), with *F*_ST_ per locus ranging from −0.0005 at locus *BOF514* to 0.2013 at *BOF305*. Pairwise multi-locus *F*_ST_ estimates for both the 40 and 32 loci datasets and the 20 samples gave similar results and indicated significant population differentiation between the most geographically distant samples (figure 1; electronic supplementary material, table S7). The Azores (*AZA*) and Western Sahara (*MOR*) samples were significantly different from each other and from all other samples. The eastern Mediterranean samples (*MTA* and *TKM*) were not significantly different from each other but were different from all other samples. There was no significant population structure between duplicate temporal replicate samples except between *PTN1* and *PTN2* in the 40 loci dataset (*F*_ST_ 0.0067). AMOVA analyses also confirmed the temporal stability of the temporal replicate samples as temporal variability (*F*_SC_) accounted for only 0.43% and 0.37% of observed variation in the 40 and 32 loci datasets, respectively (electronic supplementary material, table S8). The southern Portuguese samples (*PTS1* and *PTS2*) were significantly different to the other northeast Atlantic continental shelf samples. There was no significant difference between any of the samples collected from *BOB1* northwards; however, some structure was evident between these samples and those from the northern Spanish Shelf (*NSA1* and *NSA2*) and also northern Portuguese waters (*PTN1* and *PTN2*).

**Figure 2.**
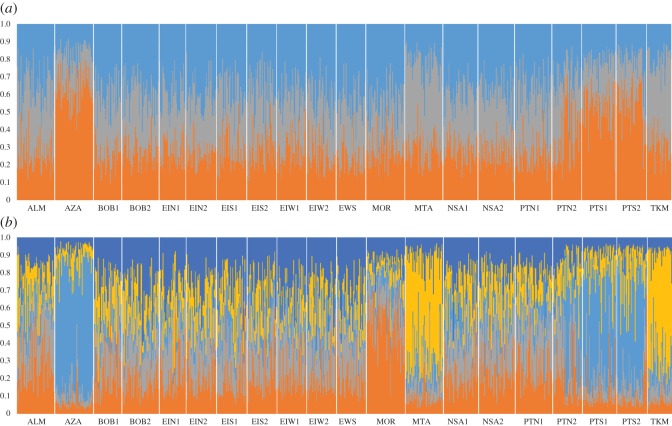
Cluster assignment of boarfish specimens from STRUCTURE analysis of (*a*) the 32 loci dataset (*K* = 3) (*b*) the 40 loci dataset (*K* = 5).

Analysis of the STRUCTURE output for the 40 loci dataset indicated that the ln *P*(*D*) reached a clear mode of *K *= 5 before decreasing (electronic supplementary material, figure S1). The output for the 32 loci dataset did not have a clear mode and in this case the delta *K* method, which indicated *K* = 3, was more appropriate. Both analyses indicated similar overall clustering patterns (figure 2*a*,*b*). The difference between the datasets was the identification of the *MOR* samples as a separate cluster and the splitting of the *AZA* and *PTS* samples into two clusters in the 40 loci dataset. The temporal replicate samples clustered together in both cases except *PTN1* and *PTN2*. The *PTN1* sample clustered together with all the samples located to its geographical north. The *PTN2* sample appeared to be a mixed sample with part similar to the northern cluster and part similar to the *PTS* cluster. Examination of the sample collection details (figure 1 and table 1) indicated that *PTN2* was collected from two different hauls: one north (*n* = 15) and one south (*n* = 27) of Cabo da Roca.

Potential genetic barriers detected in BARRIER 2.2, with more than 70% support, are illustrated and sequentially numbered in figure 1. The boundary with the greatest support separated the samples from *PTN2A* northwards from those to the geographical south. Other significant boundaries separated the clusters described later.

### 3.6. Sample pooling and clustering

As there were no significant differences in the estimated global multi-locus *F*_ST_ estimates, pattern of population structure (see also pairwise LOSITAN analysis below) or power to detect population structure between the 40 and 32 loci datasets, only the 40 loci dataset was retained for subsequent analyses. The pairwise *F*_ST_, STRUCTURE, AMOVA and BARRIER analyses indicated that there were no significant structure, no temporal variation and no genetic barriers between samples north of *BOB1*, therefore, these samples were pooled in a single northeast Atlantic sample (*NEA*). Similarly, the *NSA1*, *NSA2* and *PTN1* samples were pooled into a single sample. The *PTN2* sample was split into *PTN2A* and *PTN2B* as described above, with *PTN2B* being merged with *PTS1* and *PTS2*. The eastern Mediterranean samples (*MTA* and *TKM*) were pooled into a single *MED* sample. The *ALM*, *AZA* and *MOR* samples were retained as distinct samples. The resulting dataset consisted of eight clustered samples.

The global multi-locus *F*_ST_ for the clustered dataset was estimated at 0.0296 (0.0195–0.0417 95% CI) and pairwise *F*_ST_ estimates indicated significant population structure between all samples (table 4). The *AZA* sample showed the highest degree of genetic differentiation between it and the other samples (average *F*_ST_ = 0.0622), though it was less differentiated from the *PTN2B-PTS* sample than the other samples. The *MOR* and *MED* samples also showed a relatively high level of genetic differentiation from the other samples, average *F*_ST_ = 0.0446 and 0.0461, respectively. The lowest level of genetic differentiation was observed between the *NEA* and *NSA-PTN1* samples (*F*_ST_ = 0.0041). The level of genetic differentiation between the *NEA* and *PTN2B-PTS* (*F*_ST_ = 0.0331) was comparable with the differentiation between the *NEA* and *MOR* (*F*_ST_ = 0.0253), though both were higher than the differentiation between *NEA* and *ALM* (*F*_ST_ = 0.0099).

**Table 4.** Pairwise multi-locus *F*_ST_ estimated in MSA for clustered samples. *F*_ST_ values are above the diagonal and associated *p*-values below. All *p*-values were judged significant after sequential Bonferroni correction.

*F*_ST_	ALM	AZA	NEA	MOR	NSA-PTN1	PTN2A	PTN2B-PTS	MED
ALM		0.0759	0.0099	0.0193	0.0072	0.0138	0.0390	0.0380
AZA	0.0001		0.0669	0.0741	0.0644	0.0741	0.0116	0.0684
NEA	0.0001	0.0001		0.0268	0.0041	0.0192	0.0331	0.0303
MOR	0.0001	0.0001	0.0001		0.0253	0.0443	0.0447	0.0776
NSA-PTN1	0.0001	0.0001	0.0001	0.0001		0.0105	0.0266	0.0334
PTN2A	0.0032	0.0001	0.0001	0.0001	0.0048		0.0371	0.0357
PTN2B-PT	0.0001	0.0001	0.0001	0.0001	0.0001	0.0001		0.0391
MED	0.0001	0.0001	0.0001	0.0001	0.0001	0.0001	0.0001	

**Figure 3.**
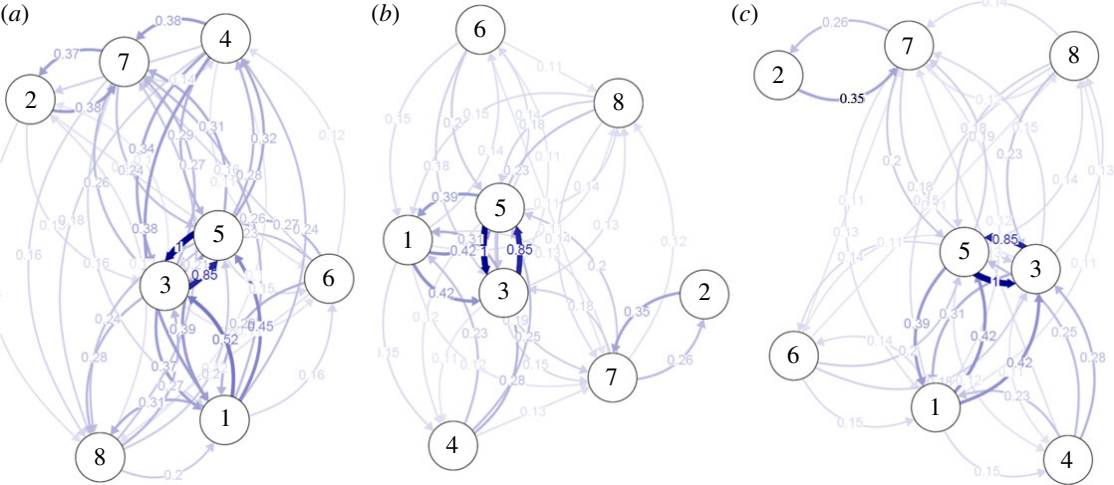
The directional relative migration networks of clustered boarfish samples from divMigrate including all relative migrations values (*a*) Jost's *D* (*b*) *G*_ST_ (*c*) *Nm*. The sample clusters are indicated by number: 1, *ALM*; 2, *AZA*; 3, *NEA*; 4, *MOR*; 5, *NSA-PTN1*; 6, *PTN2A*; 7, *PTN2B-PTS*; 8, *MED*.

The directional relative migration networks as estimated by divMigrate depicted relative migration rates between the eight sample dataset (figure 3*a*–*c*). For all three estimators (Jost's *D*, *G*_ST_, *Nm*), clusters grouped in a similar pattern to the STRUCTURE and pairwise *F*_ST_ analyses, with the *NEA* and *NSA-PTN1* grouping close together. The estimated gene flow from *NSA-PTN1* to *NEA* (1.0) was higher than the reverse (0.85) in all analyses, although it was only significantly asymmetrical in the *Nm* analysis. The *ALM* sample also exhibited a relatively high gene flow with this northern group for all three estimators. The relative gene flow was higher between *AZA* and *PTN2B-PTS* than between them and any other samples. The *MED* sample appeared relatively isolated from the other samples in all three cases. The gene flow between the *MOR* sample and the other samples varied among the estimators, being higher with *PTN2B-PTS* in the Jost's *D* analysis and higher with *NEA* in the *G*_ST_ and *Nm* analyses. The gene flow between *MOR* and *NEA* was only significantly asymmetrical in the *Nm* analysis. No further significant asymmetries were detected in the analyses.

### 3.7. Saturation and high-grading

**Figure 4.**
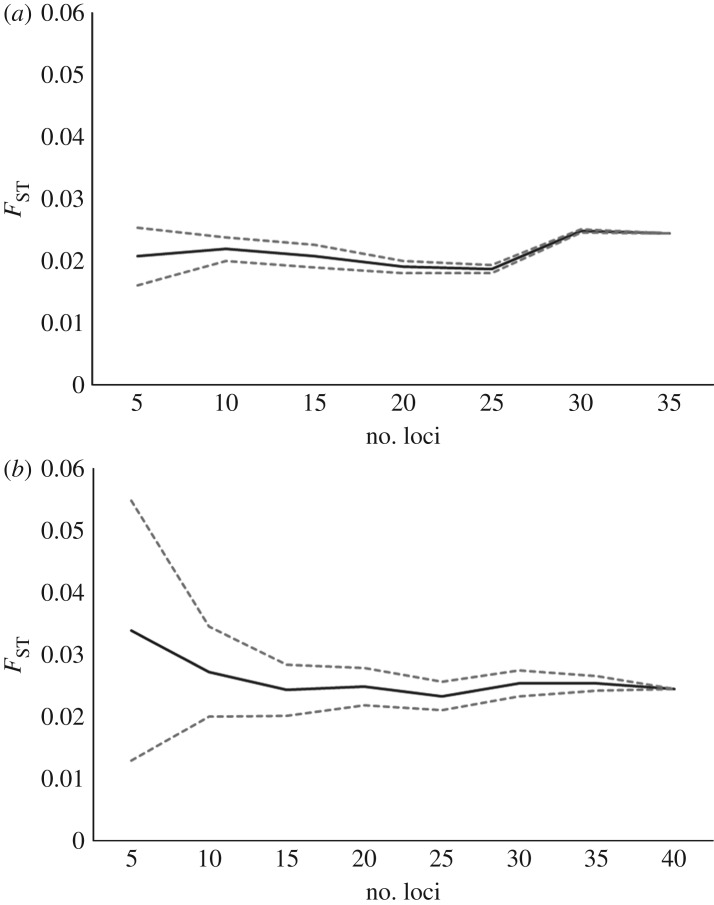
*F*_ST_ replicate sampling with (*a*) the eight outlier loci (as indicated by LOSITAN) excluded (*b*) all 40 loci included. 95% confidence interval is displayed.

Replicate sampling of loci to illustrate the effect of increasing numbers of loci on global multi-locus *F*_ST_ estimates and their variances indicated that increased number of loci reduced the variation in global multi-locus *F*_ST_ estimates (figure 4*a*,*b*). The point of diminishing return, with and without putatively outlier loci included, for increasing number of loci equated to approximately 30 loci, beyond which there was little gain in precision of *F*_ST_ estimates.

**Figure 5.**
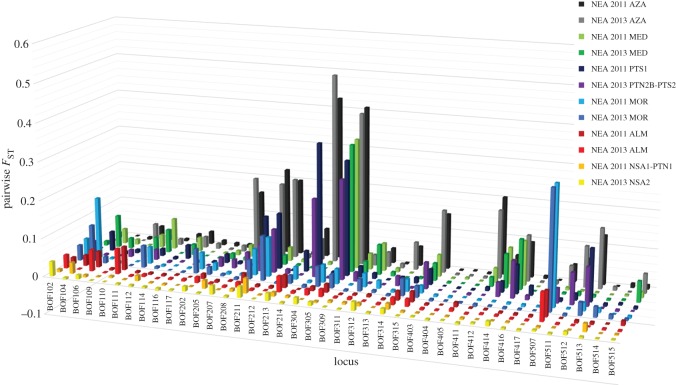
The pairwise single-locus *F*_ST_ estimates for the 2011 and 2013 *NEA* samples versus all other samples.

The pairwise single-locus *F*_ST_ estimates indicated that there was potential for high-grading loci. In general, loci that illustrated high *F*_ST_ in one temporal sample were also high in the temporal replicate sample. For example, the 2011 and 2013 *NEA* samples versus all other samples clearly shows that loci showing high *F*_ST_ in 2011 also showed similar levels of *F*_ST_ in 2013 (cf. figure 5; electronic supplementary material, table S9). In addition, it is clear that different high-graded loci should be used to differentiate between different populations. Similarly, pairwise LOSITAN runs using groupings informed by the clustering analyses indicated that of the eight potential outlier loci only three were considered outliers in multiple pairwise tests (table 5). However, these three loci were not outliers in all pairwise comparisons. Locus *BOF507* was an outlier, potentially under directional selection, in pairwise comparisons involving the *MOR* sample suggesting a geographical influence on this locus. Similarly, the locus *BOF214* was an outlier potentially under directional selection in three pairwise comparisons involving the *AZA* and an outlier under potentially balancing selection in a pairwise comparison between *NEA* and *MOR*.

**Table 5.** Results of the pairwise LOSITAN outlier analyses under the infinite allele (IA) and stepwise mutation model (SMM).

		BOF116	BOF211	BOF214	BOF305	BOF404	BOF417	BOF507	BOF514
global		IA^a^	IA + SMM	IA + SMM	IA + SMM	IA + SMM^a^	IA + SMM^a^	IA + SMM	IA^a^
pairwise comparison									
NEA	MOR			SMM^a^				IA + SMM	
NEA	NSA-PTN1								
NEA	PTN2B-PTS				IA				
NEA	MED				IA + SMM				
NEA	AZA			IA + SMM	IA + SMM				
NEA	ALM								
AZA	MOR			SMM	SMM				
AZA	NSA-PTN1				IA				
AZA	PTN2B-PTS								
AZA	MED		IA	IA + SMM					
AZA	ALM				IA + SMM				
ALM	MOR				IA				
ALM	NSA-PTN1								
ALM	PTN2B-PTS				IA + SMM				
ALM	MED				IA + SMM				
MOR	NSA-PTN1							IA + SMM	
MOR	PTN2B-PTS				IA + SMM			IA + SMM	
MOR	MED				IA			IA + SMM	
NSA-PTN1	PTN2B-PTS								
NSA-PTN1	MED								
PTN2B-PTS	MED			IA					

^a^Balancing selection.

## 4. Discussion

The current study demonstrates a rapid, cost-effective and high-throughput approach to undertaking a complete population genetics study, from marker discovery through to genotyping, in the commercially important fisheries species, boarfish, without any prior genetic knowledge of the species. The method was implemented in a ‘real’ scenario, requiring rapid and accurate identification and delineation of boarfish biological units (populations) and the robust results are directly applicable to improving the assessment and consequently management of the northeast Atlantic boarfish fishery.

The genetic analyses of boarfish revealed significant population structure across the range of boarfish. Accurate estimation of *K* in natural populations based on STRUCTURE analyses requires careful interpretation [51]; as such, the *K* estimates from the STRUCTURE analyses were only used to guide the division of the data into biological units. Pairwise *F*_ST_, AMOVA, BARRIER and STRUCTURE analyses in combination indicated support for seven biological units (populations) (figure 1 and table 4). The eastern Mediterranean samples comprised a single population and were distinct from all other samples. Similarly, the Azorean, Western Saharan and Alboran Sea samples were distinct from all others and comprised individual population units. The Alboran Sea sample appeared to show closer affinity with the *NEA* and *NSA-PTN1* samples than with the geographically closer *PTS* samples. It is unclear what the cause of this affinity is and though beyond the scope of the current study it warrants further investigation. Of particular relevance to the assessment and management of the boarfish fishery is the identification and delineation of the population structure between southern Portuguese waters and waters to the geographical north; however, repeated genetic monitoring of the boarfish in this region should be conducted to ensure the continuing validity of this delineation. A distinct and temporally stable mixing zone was evident in the waters around Cabo da Roca. The *PTN2A* sample appeared to be significantly different from all other samples; however, this sample was relatively small (table 1) and was considered to represent a mixed sample rather than a true population. There was a statistically significant but comparatively low level of genetic differentiation between the *NEA* and *NSA-PTN1* samples (table 4) indicating population structure; however, results also indicated a high level of migration between these two populations (figure 3) and a lack of barriers to gene flow between them (figure 1). The lack of significant immigration into the northeast Atlantic population from populations to the south or from insular elements (figure 3) and the strong genetic differentiation among these regions (table 4) indicate that the purported increases in abundance in the northeast Atlantic area are not the result of a recent influx from other regions. The increase in abundance is most probably the result of demographic processes within the northeast Atlantic stock [25,30]. The exact processes involved remain to be disentangled and are beyond the scope of the current study.

Exploratory analyses indicated that a high-graded panel of approximately 7–10 informative (high *F*_ST_) loci may be sufficient for the purposes of monitoring population mixing in the *NEA* region (figure 5). Some of these loci have indications of footprints of selection (table 5); however, outlier loci can serve as powerful markers for detecting population structure in marine fish species, which typically exhibit weak population structure due to large, evolutionarily young populations with high fecundity, dispersal and gene flow [64]. Therefore, they should not be discounted in favour of putatively neutral loci and both neutral and adaptive markers can be used together for investigating population structure and marine fish stock identification [6]. Analyses of both the full 40 loci dataset and the 32 loci dataset with outliers removed also revealed the same population structure, which suggests that the inclusion of loci which have indications of footprints of selection is appropriate. Further analyses of high-grading are required to establish a definitive reduced marker panel for the *NEA* stock.

The effectiveness of the shotgun–NGS-based microsatellite discovery pipeline described by Carlsson *et al*. [7] was significantly improved in the current study by using the higher capacity Illumina MiSeq platform with the Illumina V2 2 × 250 bp PE kit. Furthermore, the current study did not pool the individuals for shotgun sequencing into a single library as in Carlsson *et al*. [7]. The division of individuals from geographically isolated areas into separate libraries within the one MiSeq run enabled putative polymorphisms to be detected during microsatellite discovery, which reduced the need for testing and optimizing an excessively large panel of microsatellite loci prior to employing them in a full-scale study.

The potential of NGS-based GBS as a method for microsatellite genotyping was first demonstrated by Vartia *et al*. [22]; however, their method was not optimized for population genetics scale genotyping, used the relatively low output 454 NGS Platform and was primarily concerned with introducing and validating the method. The current study developed the method further by increasing the capacity of the approach and implementing it on a full-scale population genetics study. Amplicon sequencing on the Illumina MiSeq platform (V3 2 × 300 bp kit) with a potential yield of 25 million reads significantly increased the capacity of the method to include more loci and more individuals within a single run. The increase in the number of combinatorial barcodes from 20 (12 forward and eight reverse) in [22] to 96 (48 forward and 48 reverse) facilitates the barcoding of up to 2304 individuals within a single library and single sequencing run, though only 960 individuals were barcoded using 68 of the barcodes (32 forward and 36 reverse). Additionally, as the combinatorial barcoding method uses universal tails for barcode annealing rather than platform-specific adapters and indexes the method is not limited to a single sequencing platform. This means the method is easily transferable between platforms, with adequate read length, as new technology become available, which is in contrast to other recently reported microsatellite GBS methods [23,65], which used Illumina specific dual indexing primers.

Though the current study presents a significant advance in the field of microsatellite GBS, there are areas where it may be further optimized. An additional RNase clean-up step should be included when isolating gDNA for shotgun sequencing to remove contamination of RNA and prevent subsequent loss of sequence effort. To ensure sufficient read depth for discovery of putatively polymorphic loci, the current study relied on the high throughput of the Illumina platform. This may be improved by reducing the size of the sequenced genome by generation of reduced representation libraries (RRL) and size selection of fragments rather than random fragmentation at a set fragment length [7,13]. These steps may be particularly important for organisms with larger genomes where low read depth at individual microsatellites may inhibit the identification of polymorphic loci.

In the current study, the 21% yield of amplicon reads with two matching barcodes and two matching primers was lower than expected and significantly lower than the 40% achieved by Vartia *et al*. [22]. It should be noted though that the current study allowed for zero errors in the barcodes or primers, while Vartia *et al*. [22] allowed for two and three errors, respectively, in order to avoid a significant loss in read depth. The other largest read group categories were ‘one barcode’ (54.1%), which included amplicons with errors in one barcode, amplicons with only one barcode annealed and also barcodes with no amplicon, and ‘no barcode’ (23.6%), which included amplicons with no identifiable barcodes annealed. Thus, there may have also been below optimum incorporation of barcodes during Step 2 of the two-step PCR, possibly due to a deficit of remaining Multiplex PCR Master Mix in the reaction for this step or the subdivision of the single-nested PCR [22] into two separate steps. The yield of barcoded amplicons may also have been improved by performing a size selection on the pooled amplicon samples prior to library preparation. Further analysis and optimizations are required to improve upon these aspects, which will enable a higher throughput in future studies. Similarly, high-grading of loci and employing a reduced panel of informative loci would enable more individuals to be included in each sequencing run while still retaining sufficient read depth for accurate genotyping. If necessary, more barcodes may be readily developed from the 2167 available barcodes [42] and added to the existing 96 from the current study. If smaller studies are being conducted then the amplicons of multiple species may be pooled into a single sequencing run, thus reducing costs and increasing efficiency.

There is a long-standing debate on the relative merits of both microsatellites and SNPs for population genetics, with both having recognized advantages and limitations [5]. Ultimately, the choice of marker depends on the questions being asked and many questions in molecular ecology can be answered with a limited number of highly polymorphic markers, such as microsatellites [5]. Many of the perceived disadvantages of microsatellites are of a technical nature and, as Vartia *et al*. [22] demonstrated, they can be addressed by using a GBS and combinatorial barcoding approach. While still in its infancy microsatellite GBS is gathering momentum as evidenced by other subsequent publications [23,65], and there is significant scope for further development particularly with regard to the automation of the genotyping step [65,66]. While the field is still emerging, it is important to stress that efforts are made to develop generic approaches which are not NGS platform dependent and that will be able to rapidly evolve with emerging technology.

In summary, the current study implements a novel and high-throughput approach to undertaking a complete population genetics study that can be faster and cheaper than current approaches while offering better and more data [22,23]. The method was demonstrated in a ‘real’ scenario, on a species with no existing population genetic information, which required urgent and robust answers. These results may be used to inform the appropriate level on which to base the assessment and management of this species.

## Supplementary Material

ESM_Tables.xlsx Extra Supplementary tables for Farrell et al._Next Gen Pop Gen

## Supplementary Material

ESM Figure 1.tif - (A) Mean lnP(D) value with k = 1–20 for the 40 loci dataset. (B) Mean lnP(D) value with k = 1–20 for the 32 loci dataset

## Data accessibility

All data are provided in the manuscript and in the electronic supplementary tables.
