# Supplementary figures and images for: Next Gen Pop Gen: implementing a high-throughput approach to population genetics in boarfish (Capros aper)
Source: R Soc Open Sci. 2016 Dec 14;3(12):160651. doi: 10.1098/rsos.160651 (PMC5210689; doi:10.1098/rsos.160651)

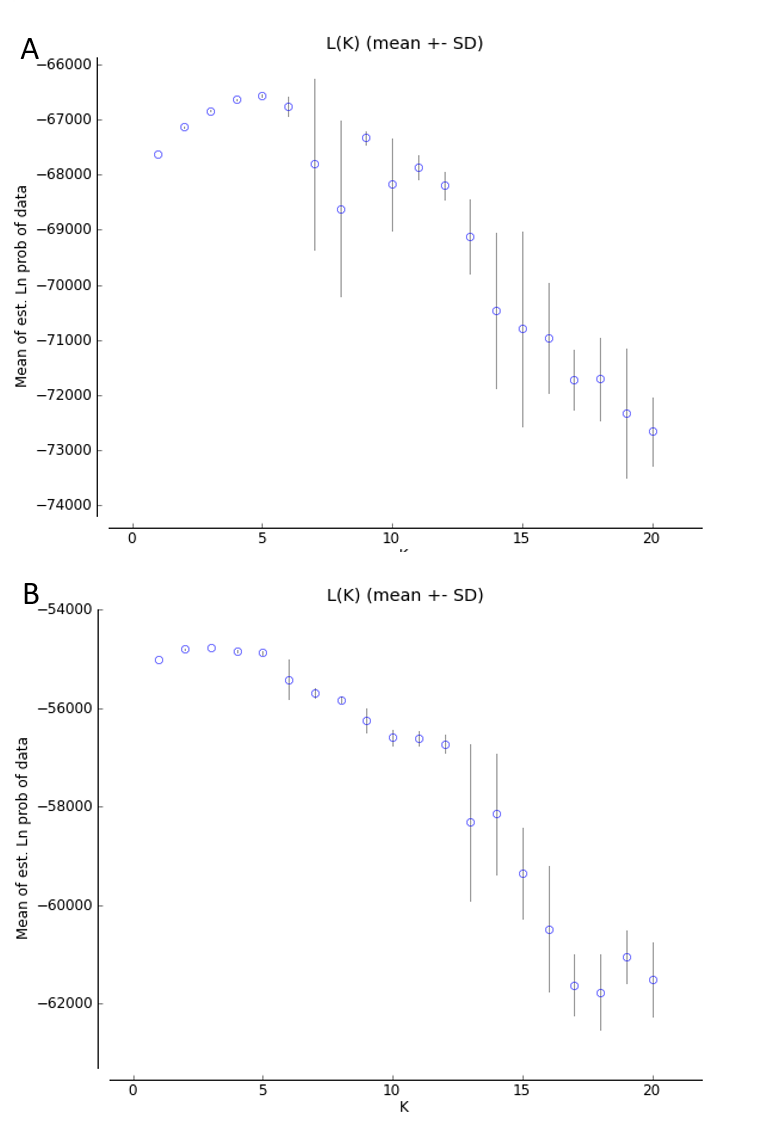

Supplement: ESM Figure 1.tif - (A) Mean lnP(D) value with k = 1–20 for the 40 loci dataset. (B) Mean lnP(D) value with k = 1–20 for the 32 loci dataset [file rsos160651supp2.tif]
